# Genetics of Monogenic Disorders of Calcium and Bone Metabolism

**DOI:** 10.1111/cen.14644

**Published:** 2021-12-21

**Authors:** Paul J Newey, Fadil M. Hannan, Abbie Wilson, Rajesh V Thakker

**Affiliations:** 1 Division of Molecular and Clinical Medicine, Ninewells Hospital & Medical School, University of Dundee, Scotland, UK, DD2 1UB; 2 Nuffield Department of Women’s and Reproductive Health, University of Oxford, UK; 3 Academic Endocrine Unit, Oxford Centre for Diabetes, Endocrinology & Metabolism (OCDEM), Churchill Hospital, University of Oxford, UK

**Keywords:** genetic testing, primary hyperparathyroidism, familial hypocalciuric hypercalcemia (FHH), familial isolated hyperparathyroidism (FIHP), multiple endocrine neoplasia, hypoparathyroidism, pseudohypoparathyroidism

## Abstract

Disorders of calcium homeostasis are the most frequent metabolic bone and mineral disease encountered by endocrinologists. These disorders usually manifest as primary hyperparathyroidism (PHPT) or hypoparathyroidism (HP), which have a monogenic aetiology in 5-10% of cases, and may occur as an isolated endocrinopathy, or as part of a complex syndrome. The recognition and diagnosis of these disorders is important to facilitate the most appropriate management of the patient, with regard to both the calcium-related phenotype and any associated clinical features, and also to allow the identification of other family members who may be at risk of disease. Genetic testing forms an important tool in the investigation of PHPT and HP patients, and is usually reserved for those deemed to be an increased risk of a monogenic disorder. However, identifying those suitable for testing requires a thorough clinical evaluation of the patient, as well as an understanding of the diversity of relevant phenotypes and their genetic basis. This review aims to provide an overview of the genetic basis of monogenic metabolic bone and mineral disorders, primarily focusing on those associated with abnormal calcium homeostasis, and aims to provide a practical guide to the implementation of genetic testing in the clinic.

## Introduction

Metabolic bone and mineral disorders represent a diverse group of conditions characterised by alterations in skeletal homeostasis and/or circulating concentrations of calcium, parathyroid hormone (PTH), phosphate and vitamin D metabolites. Many of these diseases have a genetic aetiology, either involving a single gene (i.e., monogenic disorders), larger scale genetic defects (e.g., chromosomal abnormalities including gains or losses of genetic material) or have oligogenic/polygenic inheritance involving variants in multiple genes. Genetic testing is increasingly employed to investigate patients with a potential monogenic aetiology. The appropriate use of such testing requires an understanding of the relevant phenotypes as well as the genetic basis of the respective disorders. Whilst many of the metabolic bone and mineral disorders characterised by skeletal phenotypes typically present to a range of ‘non-endocrine’ specialties (e.g., orthopaedics, specialist bone clinics and rheumatology), those primarily associated with alterations in circulating calcium (i.e., hypercalcaemia, hypocalcaemia), frequently present to the endocrinologist. Although the majority of these calcium-related disorders are acquired (i.e., sporadic), 5-10% of patients presenting with a biochemical phenotype suggestive of primary hyperparathyroidism (PHPT) or hypoparathyroidism (HP) will have a monogenic aetiology, either occurring as an isolated endocrinopathy, or as part of a wider clinical syndrome. The identification of patients with a monogenic cause is important for facilitating appropriate clinical management and allowing identification of additional family members who may be at risk of disease. This review provides an overview of monogenic disorders of bone and mineral metabolism, with a focus on the more commonly encountered disorders associated with PHPT and HP phenotypes.

## Genetic basis of monogenic calcium and metabolic bone disorders

Monogenic disorders affecting calcium and bone metabolism frequently arise from germline mutations affecting the coding region of the responsible gene and are predominantly inherited as autosomal or X-linked traits (Table 1-3).^1,2^ This includes autosomal dominant (e.g., Multiple Endocrine Neoplasia type 1 (MEN1) and type 2A (MEN2A)), autosomal recessive (e.g., Autoimmune Polyendocrine Syndrome type 1 (APS1)), X-linked dominant (e.g., X-linked hypophosphataemic rickets) and X-linked recessive (e.g., Dent disease) patterns of inheritance. In addition, mitochondrial inheritance may be observed (e.g., hypoparathyroidism associated with Kearns-Sayre syndrome), as may germline mosaicism in which a post-zygotic mutation occurs in one of the parental germ cells, resulting in an apparent autosomal recessive pattern of inheritance with multiple affected offspring of unaffected parents.^3^ Parent-of-origin effects may also occur due to genomic imprinting; for example, maternally inherited inactivating *GNAS* mutations which encodes the G-protein alpha subunit (Gαs), cause pseudohypoparathyroidism type 1A (PHP1A)/Albright’s hereditary osteodystrophy (AHO), whilst the equivalent paternally-inherited mutations cause AHO without accompanying endocrine manifestations (e.g., pseudopseudohypoparathyroidism (PPHP)).^4,5^ There may also be preferential transmission of the mutant allele (i.e., transmission ratio distortion) as reported for mother-to-offspring transmission of PHP1A-associated *GNAS* and pseudohypoparathyroidism type 1B (PHP1B)- associated *Syntaxin 16 (STX16) gene* mutations.^6,7^ Monogenic disorders may arise in the absence of a relevant family history; for example, McCune-Albright syndrome arises from a post-zygotic somatic *GNAS* mutation in early embryonic development;^8,9^ whilst many disorders may be associated with *de novo* germline mutations (e.g., ~10% of index cases with MEN1).^10,11^

The majority of monogenic disorders arise from genetic variation affecting the coding region of the responsible gene to alter gene function (e.g., gain or loss of function). This typically involves small changes in DNA sequence (e.g., single nucleotide variants (SNVs) resulting in missense or nonsense amino acid substitutions or splice site changes, or small insertions and/or deletions of nucleotides (e.g., <10 nucleotides) resulting in either in-frame of frameshift alterations in amino acid sequence. In some instances monogenic disorders results from larger scale chromosomal abnormalities (i.e., copy number variations (CNVs) due to chromosomal deletions and/or insertions), which may not be identified by direct DNA sequencing methods, but rely on alternate methods of detection (e.g., array comparative genomic hybridization (aCGH)).

Several disorders are associated with genetic heterogeneity, in which similar or overlapping clinical phenotypes may result from mutations in one of several different genes. For example, three forms of FHH are recognized due to mutations in the Calcium Sensing Receptor *(CASR)*, G-protein Subunit Alpha 11 *(GNA11)* and Adaptor Related Protein Complex 2 Subunit Sigma 1 *(AP2S1) genes*, respectively. Furthermore, opposing clinical phenotypes may arise from loss- or gain-of -function variants within the same gene. For example, loss-of-function *Glial Cells Missing Transcription Factor 2 (GCM2)* variants are associated with isolated HP, whilst activating *GCM2* variants are reported in familial isolated hyperparathyroidism (FIHP).^12,13^ Similarly, whilst inactivating *CASR* and *GNA11* mutations are associated with FHH types 1 and 2, gain of function variants in these genes result in autosomal dominant hypocalcaemia (ADH) types 1 and 2, respectively.^14,15^ Finally, the severity of phenotype may be determined by whether variants are inherited in a dominant or recessive manner. For example, autosomal recessive inheritance of *Tissue Non-specific Alkaline Phosphatase (TNSALP)* mutations are associated with severe perinatal and infantile forms of hypophosphatasia, whilst autosomal dominant *TNSALP* mutations typically result in milder later-onset forms of disease (Table 1).^16^

## Identification of Monogenic Disorders of Calcium Homeostasis in the Clinic and Utility of Genetic Testing

### History & Examination

An underlying monogenic disorder should be considered in all patients presenting with disorders of calcium homeostasis and careful clinical evaluation may alert the clinician to a hereditary cause. For example, familial PHPT frequently occurs at a young age with equal sex distribution, with ~50% of childhood onset PHPT having a monogenic aetiology (e.g., due to MEN1, neonatal severe hyperparathyroidism (NSHPT), hyperparathyroidism jaw-tumour syndrome (HPT-JT)).^17,18^ Similarly, the majority of childhood onset HP has a genetic cause either due to chromosomal abnormalities (e.g., DiGeorge type 1 (DGS1) syndrome due to 22q11.2 deletion) or monogenic aetiologies (Table 3).^19,20^ Likewise, presentations in infancy may indicate abnormalities in parathyroid development (e.g., isolated HP due to *GCM2* or *Parathyroid Hormone (PTH)* mutations) or aberrant *CASR* signalling (e.g., NSHPT).^21,22^

Evaluating previous medical records, historic laboratory results and radiological imaging may help elucidate the cause. For example, a longstanding biochemical abnormality may suggest a monogenic aetiology. Thus, in FHH1-3 and ADH1-2, the respective hyper- and hypocalcaemia is typically lifelong.^23^ Evaluating patients for previous or current manifestations of syndromic PHPT or HP disorders may provide diagnostic clues. For example, a preceding endocrine tumour (e.g., insulinoma, Cushing’s disease) in a patient with PHPT may indicate MEN1, whilst cardiac defects, or presence of other autoimmune disease in a patient HP may suggest a unifying diagnosis of DGS type 1, or autoimmune polyglandular syndrome type 1 (APS1), respectively.^19,24–26^ In some instances, the possibility of a monogenic disease will only arise after further investigation or treatment. For example, the finding of parathyroid carcinoma should raise the possibility of the HPT-JT, whilst a failure to restore normocalcaemia after parathyroid surgery may increase the likelihood of FHH.

### Family History

Establishing the patient’s family history is paramount to the clinical evaluation. However, the availability of such information is often limited due to the patient being unaware of their relative’s medical history. Likewise, a family history may be absent if affected family members (i.e., those carrying the disease-associated variant) remain undiagnosed, whilst for disorders with reduced disease penetrance, affected family members may remain asymptomatic and/or ‘disease-free’. Furthermore, when variants arise *de novo*, a relevant family history will be absent. A history of consanguinity should raise the possibility of autosomal recessive disease (e.g., hypoparathyroidism due to *GCM2* mutations), whilst specific ethnic or geographical backgrounds may increase the likelihood of known ‘founder’ mutations (e.g., APS1-associated *AIRE* mutations in Finnish and Sardinian populations).^25,27,28^ Although the family history should provide insight into the mode of inheritance, occasionally diagnostic confusion may arise. For example, both germline and somatic mosaicism have been reported in the setting of MEN1.^3,29^

### Utility of Genetic Testing

Undertaking genetic testing in individuals with a potential monogenic disorder has several potential benefits.^1,30^ Confirmation of a genetic diagnosis may not only facilitate appropriate treatment (e.g., conservative management of FHH, surgical approach for MEN1-related PHPT), but also allow the identification of associated features that are not clinically apparent (e.g., non-functioning pancreatic neuroendocrine tumours in MEN1). **In some instance a genetic diagnosis may facilitate personalised treatment strategies (e.g. use of enzyme-replacement therapy with asfotase-alfa to treat paediatric-onset hypophosphatasia, or use of the anti-FGF23 monoclonal antibody burosumab to treat children with X-linked hypophosphataemic rickets)**.^31–33^ Furthermore, establishing a genetic diagnosis may facilitate: predictive testing in asymptomatic family members; preconception genetic counselling; prenatal genetic testing; and in some settings pre-implantation genetic diagnosis.^1^ Genetic testing may also resolve diagnostic uncertainty arising from phenocopies in which a patient manifesting the clinical phenotype of a particular genetic disorder is found not to harbour the expected gene mutation. Indeed, phenocopies involving patients with clinical manifestations of MEN1, MEN2A, HPT-JT and FHH are widely reported.^34,35^ Genetic testing demonstrating an absence of a causative mutation is also of value and may provide reassurance to the patient and wider family.

## Hereditary Hypercalcaemic Disorders

Monogenic causes of hypercalcaemia are broadly divided into PHPT syndromes, those in which PHPT occurs as an isolated endocrinopathy, disorders associated with impaired CaSR signaling, and conditions associated with abnormal vitamin D metabolism (Table 2). In the endocrine clinic, MEN1 and FHH type 1 are likely to be encountered most frequently.^36^

## PHPT syndromes

### Multiple Endocrine Neoplasia Type 1 (MEN1)

#### Clinical Features

MEN1 has a reported prevalence of 1 in 30,000 individuals and is characterised by the combined occurrence of parathyroid, pituitary and pancreatic endocrine tumours. Some patients may develop additional tumours including thymic and bronchial carcinoid tumours and adrenocortical tumours.^10,11,37^ PHPT occurs with almost complete penetrance (>95% by age 50 years) and is the first manifestation of disease in ~75% of cases, occurring with a mean age of onset of ~20 years, although cases have been reported as early as 4-years of age.^26^ The biochemical features of PHPT are typically mild and patients are frequently asymptomatic. Characteristically, there is synchronous or asynchronous involvement of all 4 parathyroid glands, with histology demonstrating chief cell hyperplasia. Surgical resection is the treatment of choice although its timing and extent remain controversial.^11^ **Notably, hypercalcaemia may exacerbate gastrin secretion in those with concurrent PHPT and gastrinoma, and restoration of normocalcaemia (e.g. by successful parathyroidectomy) may improve symptoms in a proportion of patients with Zollinger-Ellison syndrome.^11^**

#### Genetics

MEN1 results from heterozygous inactivating mutations of the *MEN1* gene, which encodes the tumour suppressor protein Menin, Greater than 600 different germline *MEN1* mutations have been reported, which occur throughout the coding region.^38–41^ To date, no clear genotype-phenotype correlation has been established and in ~10% of index cases *MEN1* mutations occur *de novo*. Genetic testing employing DNA sequencing does not reveal a coding-region *MEN1* mutation in all MEN1 patients, who may: harbour genetic alterations in non-coding *MEN1* regions; have whole of partial *MEN1* gene deletions; have mutations in other genes (e.g., Multiple Endocrine Neoplasia Type 4 (MEN4) due to *Cyclin Dependent Kinase Inhibitor* 1B (*CDKN1B)* mutations); harbour somatic mosaic *MEN1* mutations; represent phenocopies; or have the chance occurrence of two or more sporadic endocrine tumours.^11,42,43^ Current MEN1 guidelines recommend *MEN1* genetic testing in patients with: a clinical diagnosis of MEN1 (i.e. 2 or more MEN1-associated tumours); multi-gland parathyroid adenomas or hyperplasia <40 years of age, recurrent hyperparathyroidism; gastrinoma or multiple pancreatic NETs; atypical expressions of MEN1 (e.g., ≥1 endocrine and ≥1 non-endocrine MEN1-related tumour); ≥1 MEN1-related manifestation with a first degree relative (FDR) with ≥1 MEN1-related tumour; and all FDRs of a family member with a known *MEN1* mutation.^11^ Current NHS England guidelines have broadly similar recommendations although suggest that patients with insulinoma or pituitary adenomas <20 years of age, or pituitary macroadenomas <30 years of age, also undergo *MEN1* genetic testing. When an index case is identified to harbour a *MEN1* mutation, cascade genetic testing should be offered to all FDRs with appropriate genetic counselling.^11^

### Multiple Endocrine Neoplasia Type 2A (MEN2A)

#### Clinical Features

MEN2A is characterised by the combined occurrence of medullary thyroid cancer (MTC) in association with phaeochromocytoma and PHPT.^44,45^ A genotype-phenotype correlation is observed such that the timing of MTC onset and likelihood of other clinical manifestations is related to the specific *Ret Proto-Oncogene (RET)* mutation. MTC is the major cause of premature mortality in MEN2A patients, and frequently presents at an advanced disease stage in index cases (median age of diagnosis 20-25 years for codon 634 mutations).^45^ PHPT occurs in ≤30% of patients with MEN2A, most commonly with codon 634 mutations, and typically presents in the 3^rd^ to 4^th^ decade, although PHPT has been reported as early as 2-years of age. The extent of parathyroid involvement is variable and may involve single or multiple glands. Surgical removal of the affected glands is recommended.^45^

#### Genetics

MEN2A results from germline activating mutations of the *RET* protooncogene, which encodes a single-pass transmembrane tyrosine kinase receptor involved in neural crest and enteric nervous system development.^46,47^ More than 50 different germline missense *RET* mutations have been reported in MEN2A patients, and of the high penetrance variants, those affecting specific cysteine residues (e.g., Cys618, Cys620, Cys634) within the cysteine-rich extracellular domain occur most commonly in European populations, although variants affecting other locations (e.g., intracellular tyrosine kinase domain) also occur.^48^ Of these, the Val804Met *RET* mutation is observed at an unexpected high frequency in the background population and appears to be associated with low disease penetrance (i.e., <5%).^30,49^ Genetic testing for MEN2A is recommended in all patients with MTC, those with a clinical diagnosis of MEN2A, or ≥1 MEN2-related endocrine tumour (e.g., phaeochromocytoma) and a FDR with ≥1 MEN2-related endocrine abnormality. ‘Prophylactic’ thyroidectomy is recommended in the majority of *RET* mutation carriers including those identified through cascade testing to minimize the risk of metastatic MTC, with the timing dependent on the risk category of *RET* mutation.

### Multiple Endocrine Neoplasia Type 4 (MEN4)

#### Clinical Features

MEN4 has a similar clinical phenotype to MEN1 and should be considered when *MEN1* genetic testing d*oes* not reveal a pathogenic mutation.^43^ To date, <20 MEN4 kindreds have been reported. PHPT occurs in >90% of patients and is usually detected at a later age compared to MEN1 (typically >40 years).^43^ Asynchronous involvement of multiple parathyroid glands may occur, similar to MEN1. Other manifestations of MEN4 include functioning and non-functioning pituitary adenomas (30-40%), pancreatic and gastrointestinal neuroendocrine tumours (5-30%), and bronchial and cervical NETs.^43^

#### Genetics

MEN4 results from germline inactivating mutations of the tumour suppressor *CDKN1B* gene, encoding the cyclin-dependent kinase inhibitor p27kip. The finding of a pathogenic *CDKN1B* mutation should prompt clinical follow up similar to that recommended for MEN1, as well as cascade testing of FDRs.

### Hyperparathyroidism Tumour Syndrome (HPT-JT)

#### Clinical features

HPT-JT is characterised by the development of parathyroid tumours in association with ossifying fibromas of the maxilla and mandible.^50–53^ Patients may develop other tumour types affecting the kidneys, uterus, thyroid, pancreas and pituitary.^51^ Parathyroid tumours occur in 65-90% of patients, typically arising in early adulthood, although may occur at <10 years of age.^51–53^ Parathyroid tumours usually occur as solitary lesions, although there may be synchronous or asynchronous involvement of multiple glands.^51,54^ The recognition of HPT-JT is important as 15-20% of patients develop parathyroid carcinoma (PC), and this is reported as early as 8 years of age.^55^ Surgical resection is recommended for parathyroid tumours due to the increased risk of PC, **although the approach varies in different centres. Thus, in some centres bilateral neck exploration with selective removal of the abnormal gland(s) is recommended, whilst in others, a focused approach, when pre-operative localisation studies have revealed single-gland involvement, is advocated.^54^** Single or multiple ossifying fibromas occur in 10-30% of patients.^51,52^

#### Genetics

HPT-JT results from germline inactivating mutations of the *Cell Division Cycle 73 (CDC73)* gene, which encodes the tumour suppressor protein, Parafibromin.^52^ More than 50 different germline *CDC73* mutations have been reported,.^51–53^ No clear genotype-phenotype correlation has been established although a ~6 fold increased risk of parathyroid carcinoma is reported in those with ‘high-impact’ germline *CDC73* mutations (i.e., those predicted to cause marked conformational disruption or loss of parafibromin expression).^56^ Genetic testing for *CDC73* mutations is recommended in: patients with the HPT-JT phenotype; all patients with PC; those presenting with PHPT at a young age without an *MEN1* mutation; patients with PHPT and a family history suggestive of familial isolated hyperparathyroidism (FIHP); and should also be considered in those with atypical features on histology or multi-gland disease.^52,57^

## Non-Syndromic Monogenic PHPT

### Familial Isolated Hyperparathyroidism (FIHP)

#### Clinical features

FIHP refers to autosomal dominant PHPT occurring as an isolated endocrinopathy, in the absence of clinical features associated with the known PHPT syndromes (e.g., MEN1, MEN4, HPT-JT).^58^ Distinguishing FIHP from syndromic PHPT such as MEN1 is difficult as PHPT is frequently the first manifestation of disease. Furthermore, FIHP may be an allelic variant of disorders such as MEN1 and for practical purposes individuals with FIHP harbouring *MEN1* mutations should be followed up and monitored as per the guidelines for MEN1 (similarly if patients with FIHP have *CDC73* mutations, clinical follow up should follow those recommended for HPT-JT). Heterozygous and homozygous inactivating *CASR* mutations are occasionally reported in kindreds with apparent FIHP (i.e., with clinical, biochemical and histological features more typical of PHPT than FHH), in whom parathyroid surgery has improved hypercalcaemia.^59–61^ However, the finding of an inactivating *CASR* mutation in a kindred with hereditary hypercalcaemia usually indicates a diagnosis of FHH (see below). The genetic basis of the majority of FIHP cases remains unexplained, although 15-20% of kindreds are reported to harbour *GCM2* mutations, and may have an increased prevalence of multi-gland parathyroid disease, lesser rates of surgical cure, and increased risk of parathyroid carcinoma in such families.^13,62–64^ The majority of genetically unexplained FIHP kindreds have low numbers of affected individuals,^58^ suggesting either a low penetrance genetic aetiology, or the chance occurrence of sporadic cases within a family. Germline variants in several of the cyclin-dependent kinase inhibitor genes (e.g., *CDKN1A, CDKN1B, CDKN2B, CDKN2C)* have been reported in apparently sporadic parathyroid adenomas in the absence of a relevant family history such that their role as potential PHPT predisposition genes remains uncertain.^65,66^

#### Genetics

*GCM2* encodes the parathyroid-specific transcription factor GCMb. The majority of FIHP-associated *GCM2* mutations are missense variants associated with enhanced transcriptional activity and occur within a C-terminal conserved inhibitory domain. An increased prevalence of germline missense *GCM2* variants is also reported in patients with apparently sporadic parathyroid adenomas, whilst certain FIHP and PHPT-associated *GCM2* variants are enriched in individuals of specific ethnic background (e.g., Ashkenazi Jewish), and are likely associated with low disease penetrance.^64,67^ Genetic testing for each of the hereditary PHPT syndromes, FHH types 1-3, and *GCM2* mutations should be considered in those with apparent FIHP to resolve diagnostic confusion (Figure 2).

## Familial Hypocalciuric Hypercalcaemia (FHH) & Neonatal Severe Hyperparathyroidism (NSHPT)

### Clinical features

**Figure 1:**
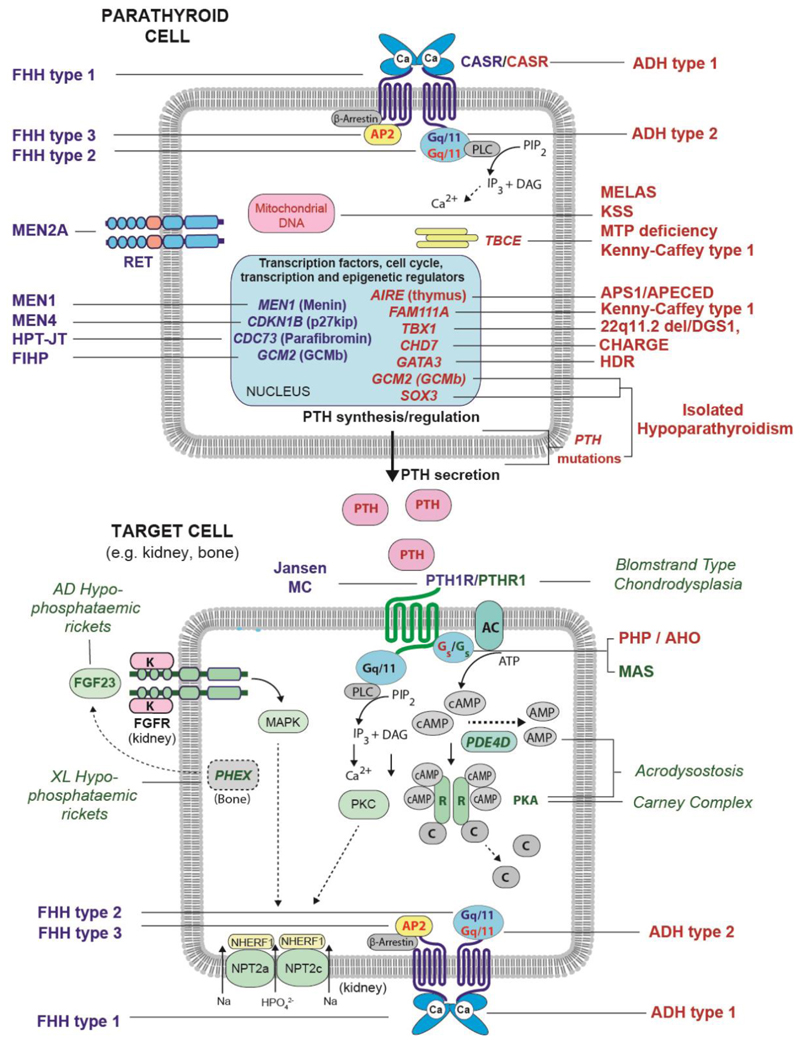
Schematic highlighting molecular components associated with monogenic disorders of calcium and bone metabolism. Alterations in extracellular calcium are detected by the calcium-sensing receptor (CaSR), which is expressed in parathyroid, kidney and bone cells. The CaSR signals via the G_q/11_ proteins to stimulate phospholipase C (PLC), which catalyses the hydrolysis of phosphoinositide (PIP_2_) to inositol triphosphate (IP_3_), thereby increasing intracellular calcium (Ca^2+^_i_), and diacylglycerol (DAG). Expression of CaSR is also regulated by the AP2 adaptor complex (AP2) which is involved in clathrin-mediated endocytosis. In parathyroid cells, CaSR activation decreases PTH secretion. Parathyroid gland development and function are regulated by genes encoding: transcription factors (e.g., *GCM2, GATA3, TBX1*), members of epigenetic regulatory complexes (e.g., *MEN1, CDC73*), and mitochondrial and cytoskeletal proteins. Parathyroid cells express other receptors (e.g., *RET*) that may influence their proliferation/behaviour. The PTH1 receptor is abundantly expressed in kidney and bone where it regulates calcium and phosphate homeostasis. Binding of PTH to the PTH1 receptor results in activation of two second messenger pathways: Gs^-^ dependent cAMP/Protein Kinase A (PKA) and G_q/11_-dependent IP3/Protein Kinase C (PKC). The generation of cAMP results in binding to the PKA regulatory subunits (R) and release of catalytic subunits (C), facilitating serine-threonine phosphorylation of downstream target proteins. cAMP levels are also regulated by degradation, mediated by phosphodiesterase enzymes (e.g., *PDE4D*). Renal phosphate homeostasis is predominantly regulated in the proximal renal tubule by two apically expressed Na/Pi co-transporters. Activation of the PTH1 receptor and FGF receptor inhibit phosphate reabsorption. In the latter setting, the ligand FGF23, whose expression is regulated by the endopeptidase PHEX, binds to the FGF receptor in the presence of the membrane bound klotho (K) co-receptor, to activate MAPK and downstream phosphorylation of the Na^+^-H^+^ exchanger regulatory factor 1 (NHERF1) and internalization of the NPT2a and NPT2c proteins. Mutations in several genes involved in parathyroid gland development and function, and/or those involved in the activities of PTH-dependent target tissues (e.g., kidney, bone) are associated with monogenic disorders of calcium and bone metabolism. In this schematic, disorders manifesting HP/hypocalcaemia are highlighted in bold red font to the right of the figure, those associated with PHPT/hypercalcaemia are shown in bold blue font to the left of the figure, whilst relevant disorders of bone/mineral metabolism not typically associated with hypo- or hypercalcaemia calcium are shown in italic green font (both sides of schematic). Additional details of each of these disorders is provided in Tables 1-3.

FHH is a genetically heterogeneous autosomal dominant disorder characterised by lifelong non-progressive mild to moderate hypercalcaemia, mild hypermagnesaemia, normal or elevated PTH concentrations and low urinary calcium excretion (e.g., calcium creatinine clearance ratio (CCCR) of <0.01,.^18,23,68,69^ Three distinct variants of FHH are reported, designated FHH types 1-3, due to loss of function mutations in *CASR, GNA11*, and *AP2S1*, respectively (Figure 1).^23,68^ The majority of patients with FHH are asymptomatic, although patients with FHH type 3 are reported to have a higher serum calcium and reduced urinary calcium excretion compared to FHH types 1 and 2, and have an increased risk of hypercalcaemic symptoms, reduced bone mineral density, and neurodevelopmental abnormalities.^23,70^ Most FHH patients do not require treatment, although cinacalcet has been reported to improve biochemical parameters in symptomatic patients with FHH types 1-3.^18,23,69,71,72^ Although the parathyroid glands in FHH may be enlarged, parathyroid surgery does not ameliorate the hypercalcaemia, and should be avoided.^50,69^ The offspring of parents with FHH are at risk of hyper- and hypocalcaemia in the neonatal period. For example, infants who inherit a paternal FHH mutation may manifest marked hypercalcaemia, whereas the unaffected offspring of mothers with FHH may manifest transient neonatal hypoparathyroidism. ^73 18,74^

### Genetics

**Figure 2:**
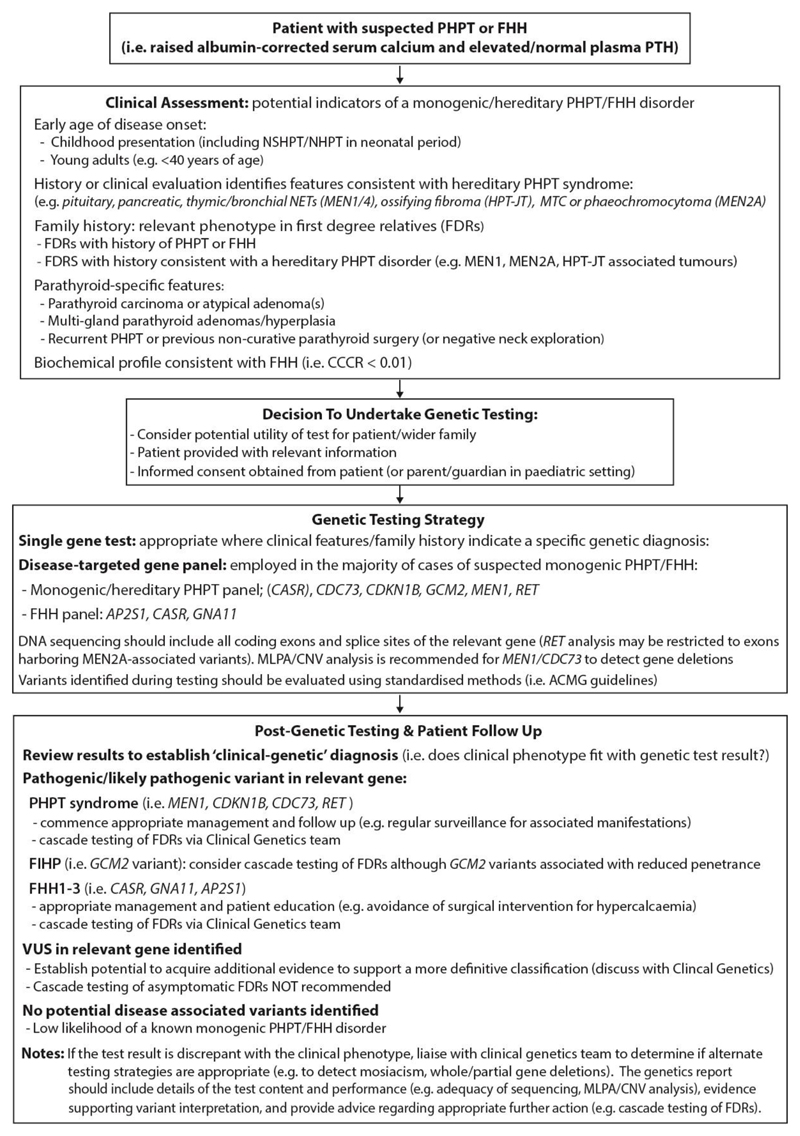
Clinical approach to establishing a genetic diagnosis in patients with PHPT/FHH A monogenic aetiology should be considered in all patients presenting with the biochemical phenotype of PHPT and/or FHH. Identifying patients in whom genetic testing is appropriate is dependent on a thorough clinical evaluation, including acquisition of relevant past medical history and family history. Specific features identified during investigation or treatment may suggest a genetic aetiology (e.g., multi-gland parathyroid hyperplasia, atypical parathyroid adenoma or parathyroid carcinoma). Specific indications for testing and the strategy employed for genetic testing (i.e., single gene testing vs disease-targeted gene panel) will depend on the clinical presentation and on local and national protocols. In most settings, disease-targeted gene panels are employed and given the overlap in biochemical phenotypes, simultaneous/combined evaluation of PHPT and FHH genes may be appropriate. Multiplex ligation-dependent probe amplification (MLPA) and/or copy number analysis should be considered for genes in which whole or partial gene deletions may result in disease (e.g., *MEN1, CDC73*). The interpretation of genetic variants should follow standardized processes (i.e., adhering to ACMG guidelines) classifying variants into one of five classes: pathogenic, likely pathogenic, benign, likely benign or variant of uncertain significance (VUS). However, this molecular classification does not equate to a clinical diagnosis and the test result should be incorporated into the overall clinical assessment of the patient, thereby achieving a ‘clinical-genetic’ diagnosis. Adjustment to ACMG guidance has been proposed for specific disorders (e.g., MEN1) to improve diagnostic accuracy.^111^ When there is uncertainty over the clinical-genetic diagnosis, discussion with the genetics team should aim to determine whether additional support for a particular diagnosis can be established. Likewise, if the clinical phenotype strongly points to a particular genetic diagnosis, but initial genetic testing does not reveal a causative mutation, it may be appropriate to consider alternate testing strategies/platforms. When molecular classification identifies a VUS, additional evidence to support a more categorical classification should be sought where possible (e.g., testing of further affected family members, additional *in vitro/in silico* functional analysis). It should be noted that variant classification may change over time such that periodic re-evaluation of variants is recommended. Where likely pathogenic/pathogenic variants associated are identified, predictive genetic testing of ‘at-risk’ family members is usually recommended (under the remit of the clinical genetics team with appropriate genetic counselling). Abbreviations: CNV, copy number variant; FDRs, first-degree relatives; MLPA, multiplex ligation-dependent probe amplification

FHH1 accounts for ~65% of all FHH cases with >150 different loss-of-function *CASR* mutations reported, and a recent population-based study indicating a genetic prevalence of ~75/100,000. ^14,15,68,75^ FHH2 is the least common form of FHH due to inactivating mutations in *GNA11* which encodes Gα11, a component of the heterotrimeric G-protein complex associated with CaSR signalling (Figure 1).^23^.^23,71,76,77^ FHH3 results from germline inactivating mutations affecting codon 15 of the *AP2S1* gene which encode the adaptor protein 2 sigma (AP2σ) subunit, which is involved in clathrin-mediated endocytosis.^23,78^ Genetic testing for FHH types 1-3 should be considered in all patients with a suggestive biochemical phenotype (i.e., raised serum calcium, normal/raised PTH, and hypocalciuria) (Figure 2).

### Neonatal Severe Hyperparathyroidism (NSHPT)

NSHPT presents in the first few weeks of life with severe hypercalcaemia (serum calcium typically 3.0-6.0mmol/l), elevated PTH, skeletal demineralization causing fracture, respiratory distress, and if untreated is frequently fatal by 3-6 months.^18,23,50,68^ NSHPT most commonly results from bi-allelic (i.e., homozygous or compound heterozygous) loss-of-function *CASR* variants, although may also occur with some heterozygous *CASR* mutations and result in less severe hypercalcaemia which may improve over time to a phenotype consistent with FHH1.^18,74,79^ NSHPT is typically treated with urgent parathyroidectomy, although bisphosphonates and/or cinacalcet may be used to control hypercalcaemia pre-operatively.^79^

Several additional rare genetic disorders may be associated with hypercalcaemia, and often present in the neonatal and paediatric settings.^18,80^ Details of these disorders are provided in Table 2.

## Hereditary Hypocalcaemic Disorders

The monogenic causes of hypocalcaemia/HP may be divided into those in which it occurs as part of a developmental or autoimmune syndrome, and those in which it is an apparent isolated feature. Of these, DGS type 1 and ADH1 are the most frequently encountered.

## Syndromes Associated with HP

### DiGeorge Syndrome/22q11.2 syndrome

#### Clinical Features

DGS affects ~1 in 4000 live births and accounts for up to 60% of cases of familial or idiopathic hypoparathyroidism in children.^81^ It is characterised by cardiac outflow tract malformations, facial dysmorphia, palatal dysfunction, hypoparathyroidism and immune deficiency related to thymic hypoplasia, although the severity of clinical features varies markedly.^19,82^ The HP phenotype may present in the neonatal period with symptomatic hypocalcaemia (e.g., seizures, laryngospasm), or at a later stage (e.g., during adolescence or adulthood).^82^

#### Genetics

The majority of DGS cases are sporadic resulting from a *de novo* heterozygous 0.25-3Mb microdeletion involving chromosome 22q11.2 (DGS type 1), although it is inherited from an affected parent in ~10% of cases.^19,83^ Although the microdeletion typically results in the loss of 30-40 genes, the majority of DGS type 1 clinical features are thought to be due to loss of the *T-Box Transcription Factor* 1 *(TBX1)* gene, involved in parathyroid and thymus development.^19,84^A more severe DGS phenotype with marked cognitive impairment is reported in association with a chromosomal deletion on chromosome 10p (DGS type 2) and may be due to loss of the *NEBL* gene.^85^ Genetic testing for DGS is indicated in those with relevant clinical features including HP in infancy or childhood.

### Autoimmune Polyendocrine Syndrome Type 1 (APS1)

#### Clinical Features

APS1, also referred to as the autoimmune polyendocrinopathy-candidiasis-ectodermal dystrophy (APECED) syndrome, has a prevalence of ~1:100,000, and is characterised by immune deficiency and a constellation of autoimmune endocrine and non-endocrine disorders.^25,86^ APS1 is classically defined as ≥2 of the triad of HP, Addison’s disease and mucocutaneous candidiasis. It is also associated with type 1 diabetes, growth hormone deficiency, primary gonadal failure, hypothyroidism, alopecia, urticaria, pernicious anaemia, chronic active hepatitis and vitiligo. APS1 is more frequent in specific geographical regions including Finnish, Iranian Jewish, and Sardinian populations.

#### Genetics

APS1 results from mutations of the *AIRE* gene, which encodes the autoimmune regulator protein, that is expressed in thymic medullary epithelial cells and regulates the elimination of organ-specific auto-reactive T-cells thereby promoting immunological tolerance to self-antigens.^27,87,88^

### Miscellaneous Syndromic HP disorders

The Hypoparathyroidism, Deafness and Renal Anomalies Syndrome (HDR) is a rare disorder characterised by HP in association with bilateral sensorineural hearing loss and renal abnormalities (e.g., bilateral cysts).^24^ HDR is due inactivating mutations of *GATA3*, which encodes a dual zinc finger transcription factor involved in the embryonic development of the common parathyroid-thymus primordia and regulation of PTH expression.^24^ Three mitochondrial disorders are occasionally associated with HP and include the Kearns-Sayre syndrome (KSS), MELAS syndrome and mitochondrial trifunctional protein (MTP) deficiency syndrome (Table 3).^81,89,90^ Details of additional rare syndromic HP disorders are provided in Table 3.

## Isolated Hypoparathyroidism

Isolated HP may be inherited as an autosomal recessive, autosomal dominant or X-linked recessive disorder. Thus, mutations in the *GCM2* gene are associated with autosomal recessive and dominant forms of isolated HP and may present with severe hypocalcaemia and low/undetectable PTH concentrations.^91,92^ The *GCM2* mutations causing autosomal recessive HP impair nuclear localization, DNA binding and/or transcriptional activity of the encoded GCMb transcription factor,^91^ whilst the heterozygous *GCM2* mutations causing autosomal dominant HP result in a truncated protein, which inhibits the wild-type GCMb protein (i.e., dominant-negative effect).^92^ Rarely, autosomal dominant and recessive forms of HP may also result from inactivating *PTH* mutations,^20,21,93–95^ whilst an ultra-rare X-linked recessive form of HP is reported due to a deletion-insertion involving chromosomes 2p25 and Xq27 (Table 3).^96^

## Autosomal Dominant Hypocalcaemia (ADH)

### Clinical Features

ADH comprises two genetically distinct forms, designated ADH types 1 and 2, which result from germline activating mutations of the *CASR* and *GNA11* genes, respectively.^15,23,68,97,98^ ADH1, accounts for most cases, and is characterised by mild to moderate hypocalcaemia in association with hypomagnesaemia, hyperphosphataemia and inappropriately low PTH concentrations.^15,23,68^ Around 50% of patients develop paraesthesia, muscle cramps or seizures.^99,100^ Patients with ADH1 have hypercalciuria and may be at risk of nephrocalcinosis or renal stones, particularly when treated with calcium **and/or vitamin D analogues (e.g. alfacalcidol, calcitriol)**.^23,68^ **Small clinical studies have reported that recombinant PTH is effective for treating symptomatic ADH1 patients without increasing urine calcium excretion.^100,101^ Pre-clinical and clinical studies have also indicated the potential utility of calcilytic drugs, which are CaSR negative allosteric modulators, as targeted therapies for ADH1.^98,102–104^** Patients with ADH1 may also exhibit ectopic calcification, and some develop a Bartter syndrome, characterized by renal salt wasting leading to volume depletion, hyper-reninaemic hyperaldosteronism and hypokalaemic alkalosis.^60^ Only a few kindreds with ADH2 have been reported and these patients have similar biochemical features to ADH1, although appear to have a milder urinary phenotype with less hypercalciuria. ADH2 patients are often symptomatic, whilst short stature is reported to affect some kindreds.^23,77,97^

### Genetics

**Figure 3:**
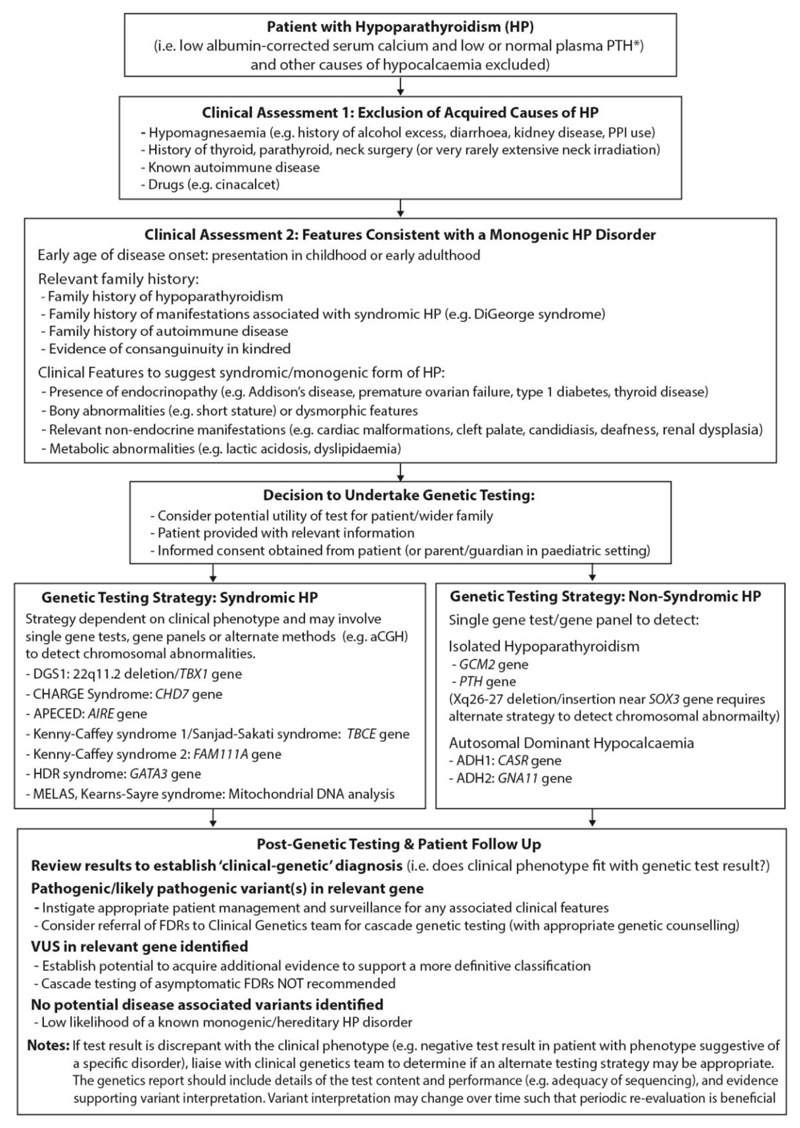
Clinical approach to establishing a genetic diagnosis in patients with Hypoparathyroidism (HP) The initial evaluation of patients with biochemical evidence of HP should aim to establish if there is an acquired cause. Once such causes have been excluded the likelihood of a genetic cause will be determined by age of onset, relevant family history, and clinical features associated with syndromic/monogenic forms of HP. The genetic testing strategy is determined by the clinical phenotype and divided according to whether a syndromic or non-syndromic cause is suspected. For the syndromic HP causes, genetic testing is targeted to the specific disorder (e.g., aCGH for DGS type 1 to detect a 22q11.2 deletion). For non-syndromic causes, which include isolated HP and ADH types 1 and 2, a disease targeted gene panel may be appropriate (i.e., including *CASR, GNA11, PTH*, and *GCMB* genes). Alternate methods may be required in instances where the genetic aetiology remains unclear (e.g., detection of an Xq26-27 deletion). The genetic confirmation of syndromic HP should facilitate investigation and management of any associated clinical features in the patient and establish whether genetic testing of other family members is indicated. Likewise, establishing a genetic diagnosis of isolated HP or ADH type 1 or 2 not only allows appropriate management of the condition but facilitates cascade testing of first-degree relatives (FDRs). * in patients with pseudohypoparathyroidism, the plasma PTH is elevated.

To date, ≥100 ADH1-associated *CASR* mutations have been reported whereas <10 ADH2-associated *GNA11* mutations have been reported.^23,77,97^ Genetic testing for ADH should be considered in all those with isolated idiopathic HP (Figure 3).

## Pseudohypoparathyroidism (PHP)/Albright’s Hereditary Osteodystrophy (AHO)

### Clinical Features

*PHP* arises from resistance to the actions of PTH, primarily in the renal proximal tubule, resulting in hypocalcaemia, hyperphosphataemia, and elevated PTH.^4,5,105,106^ Two main forms of PHP are described, PHP types 1A (PHP1A) and 1B (PHP1B). Patients with PHP1A manifest multi-hormone resistance (e.g., resistance to TSH, LH/FSH, GHRH), obesity and features of AHO, which include short stature, brachydactyly, subcutaneous calcification, and round facies.^4,5^ In contrast, PHP1B patients do not typically have multi-hormone resistance or AHO.

### Genetics

PHP primarily results from mutations affecting the *GNAS* locus, which encodes and regulates the expression of Gαs, that forms part of a heterotrimeric G-protein complex utilized by G-protein coupled receptors (GPCRs) to stimulate cAMP synthesis (Figure 1).^4,5,107,108^ *GNAS* is a complex imprinted locus, with maternally inherited heterozygous inactivating *GNAS* mutations causing PHP1A.^107,108^ In contrast, the equivalent paternally inherited *GNAS* mutations cause the related disorder of PPHP, characterised by the AHO phenotype, in the absence of PTH resistance.^107,108^ Most PHP1A/PPHP mutations affect the *GNAS* coding-region and inactivate Gαs. Abnormalities of the upstream region of the *GNAS* locus or of genes or transcripts within the *GNAS* cluster (i.e., *STX16, NESP55* and *NESPAS)*, which affect *GNAS* methylation, are reported in PHP1B.^107,108^ Genetic testing for patients with the AHO phenotype (i.e., PHP1A/PPHP) should initially involve *GNAS* sequencing as this reveals coding-region mutations in ~70% of patients, whilst testing for PHP1B may include methylation analysis of the exon 1A differentially methylated region (DMR) and/or evaluation for *STX16, NESP55* or *NESPAS* gene deletions.^4,5,107,108^

## Genetic Testing Workflow

Genetic testing should be considered in patients with disorders of calcium and bone metabolism where there is an increased likelihood of a monogenic aetiology, and where identifying such a cause will facilitate improved health outcomes in the patient and/or wider family. Genetic testing workflows for index cases presenting with hypercalcaemic and hypocalcaemia phenotypes are shown in Figures 2 and 3. Unless there is compelling reason to undertake single gene testing (i.e., clinical features or family history are suggestive of a specific disorder), the use of disease-targeted gene panels is increasingly recommended. All testing should be undertaken in accredited laboratories and variants evaluated using standardized methods (i.e., American College of Medical Genetics and Genomics (ACMG) guidelines).^109^ It should be noted that variant classification depends on the accuracy of available evidence at the time of assessment. The identification of variants of uncertain significance (VUS) pose a particular challenge, and where possible, additional information should be sought to facilitate a more definitive classification. Furthermore, the likelihood of detecting VUSs increases as the gene panel content (i.e., number of genes) increases.^30^ In addition, the genetic result should be interpreted in the clinical context of the patient. Thus, if the result is incongruent with the patient’s phenotype, further clinical and/or genetic assessment may be required.^1^ For example, a change in genetic testing platform may yield the diagnosis as illustrated by the identification of *MEN1* mutations using next generation sequencing in patients in whom previous *MEN1* sequencing had not identified a causative mutation.^29,35^ Finally, following the identification of a pathogenic/likely pathogenic variant, predictive testing of family members is warranted with the provision of relevant genetic counselling.

## Conclusions

Monogenic disorders of calcium homeostasis mainly affect parathyroid gland function, thereby resulting in isolated or syndromic forms of PHPT or HP. A thorough clinical evaluation is essential for selecting patients for genetic testing. The recognition of patients with a monogenic aetiology is important to guide appropriate management, and also to identify family members who may be at risk of disease. The genetic testing strategy is dependent on the clinical presentation and mode of inheritance, and increasingly involves analysis of disease-targeted gene panels.

## Figures and Tables

**Table 1:** Examples of Monogenic Disorders of Bone Metabolism Not Typically Associated with Abnormal Calcium Homeostasis

Hereditary Metabolic Bone Disease^a^	Estimated Prevalence	Gene	Chrom. Pos	Inherit.	Variant type	Genetic testing strategy
**Disorders of Bone and Joint** ^a^						
*Osteogenesis Imperfecta^b^*						
Osteogenesis Imperfecta (OI) Types I-IV	~1/15-20,000	*COL1A1*	17q21.33	AD (Mos)^c^	LOF, ms	Single Gene/Gene Panel
		*COL1A2*	7q21.3	AD (Mos)^c^	LOF, ms, del	Single Gene/Gene Panel
Osteogenesis Imperfecta (OI) Type VII	Rare (<1/million)	*CRTAP*	3p22.3	AR	LOF	Single Gene/Gene Panel
Osteogenesis Imperfecta (OI) Type VIII	Rare (<1/million)	*P3H1*	1p34.2	AR	LOF	Single Gene/Gene Panel
Osteogenesis Imperfecta (OI) Type XV	Rare (<1/million)	*WNT1*	12q13.12	AR	LOF	Single Gene/Gene Panel
*Disorders characterised by Epiphyseal, Metaphyseal or Chondro-dysplasia*						
Multiple Epiphyseal Dysplasia (MED) Types 1^d^	~ 1/10,000	*COMP*	19p13.11	AD	ms, del	Gene Panel
Multiple Epiphyseal Dysplasia (MED) Type 4^d^	~1/10,000	*SLC26A2*	5q32	AR	LOF, ms	Single Gene/Gene Panel
Jansen Type Metaphyseal Chondrodysplasia	Rare	*PTH1R*	3p21.31	AD	ms	Single Gene/Gene Panel
Blomstrand Type Chondrodysplasia	Rare	*PTH1R*	3p21.31	AR	LOF, ms	Single Gene/Gene Panel
Stickler Syndrome type 1	~ 1/7,500	*COL2A1*	12q13.11	AD	LOF	Single Gene/Gene Panel
Stickler Syndrome type 2	~1/80,000	*COL11A1*	1p21.1	AD	ms, del	Single Gene/Gene Panel
Spondylo-epiphyseal dysplasia tarda, X-linked	~1/150,000	*TRAPPC2 (SEDL)*	Xp22.2	XLR	LOF, del	Single Gene/Gene Panel
*Disorders with Increased BMD/Sclerosis*						
Autosomal dominant osteopetrosis/osteosclerosis	Unknown	*LRP5*	11q13.2	AD	ms	Single Gene/Gene Panel
‘High bone mass’ (Worth-type endosteal hyperostosis)	Rare (<1/million)	*LRP5*	11q13.2	AD	ms	Single Gene/Gene Panel
Sclerosteosis type 1	Rare	*SOST*	17q21.31	AR	LOF	Single Gene/Gene Panel
van Buchem disease	Rare	*SOST*	17q21.31	AR	del	Single Gene/Gene Panel
Pyle disease	Rare	*SFRP4*	7p14.1	AR	LOF, del	Single Gene/Gene Panel
Juvenile Paget disease	Rare	*TNFRSF11B*	8q24.12	AR	LOF, ms, del	Single Gene/Gene Panel
*Disorders with Reduced BMD/Osteolysis*						
Osteoporosis-Pseudoglioma syndrome	~1/2,000,000	*LRP5*	11q13.2	AR	LOF, ms	Single Gene/Gene Panel
Early-onset osteoporosis	Unknown	*WNT1*	12q13.12	AD	ms	Single Gene/Gene Panel
Familial expansile osteolysis	Rare	*TNFRSF11A*	18q21.33	AD	dup	Del/dup analysis
**Mineralisation/Vitamin D and Renal Disorders**						
Autosomal Dominant Hypophosphataemic Rickets	~ 1/20,000	*FGF23*	12p13.32	AD	ms	Single Gene/Gene Panel
X-linked Hypophosphataemic Rickets	~1/20,000	*PHEX*	Xp22.11	XLD	LOF, ms	Single Gene/Gene Panel
Autosomal Recessive Hypophosphataemic Rickets Type 1	Rare	*DMP1*	4q22.1	AR	LOF	Single Gene/Gene Panel
Autosomal Recessive Hypophosphataemic Rickets Type 2	Rare	*ENPP1*	6q23.2	AR	LOF, ms	Single Gene/Gene Panel
Vitamin D-dependent rickets, Type 1	<1/200,000	*CYP27B1*	12q14.1	AR	LOF, ms	Single Gene/Gene Panel
Vitamin D-dependent rickets, Type 2	<1/200/000	*VDR*	12q13.11	AR	LOF, ms	Single Gene/Gene Panel
Dent disease type 1	Rare	*CLCN5*	Xp11.23	XLR	LOF, ms	Single Gene/Gene Panel
Dent disease type 2	Rare	*OCRL*	Xq26.1	XLR	LOF, ms	Single Gene/Gene Panel
**Endocrine Neoplasia Disorders**						
Neurofibromatosis type 1 (NF1)	~ 1/3,000	*NF1*	17q11.2	AD (Mos)^c^	LOF, ms, del,	Single Gene/Gene Panel
Carney Complex	Rare	*PRKAR1A*	17q24.2	AD	LOF, ms, del	Single Gene/Gene Panel/aCGH
McCune-Albright Syndrome (MAS)/Polyostotic fibrous dysplasia	1/100,000-1/million	*GNAS*	20q13.32	PZ Som	ms (PZ som)	Single Gene/Gene Panel

a There is a great diversity of genetic skeletal disorders; a 2019 classification included 469 different conditions divided into 42 different groups, describing variants in 437 different genes.^110^

b Osteogenesis imperfecta (OI) is a genetically heterogeneous disorder with a large number of subtypes (i.e. Types I – XVIII). OI Types I-V are inherited in an autosomal dominant manner, whilst OI Types VI-XVIII have autosomal recessive inheritance. OI due to mutations in *COL1A1* and *COL1A2* account for >90% of cases.

c Some autosomal disorders may occur in the context of mosaicism, either arising as post-zygotic somatic mosaicism in foetal development or as germline mosaicism in an apparently unaffected parent.

d The Multiple Epiphyseal Dysplasias are a genetically heterogeneous group of disorders characterised by abnormal development of the epiphyses of the appendicular skeleton. At least 10 different disorders are described with either autosomal dominant or recessive patterns of inheritance (MED types 1 and 4, shown above).

Loss of function (LOF) variants include nonsense mutations (i.e., resulting from a SNV introducing a premature stop codon), small insertions or deletions (indels) typically resulting in frameshift alterations, and those affected canonical splice sites resulting in aberrant transcript processing. Abbreviations: aCGH, array comparative genomic hybridisation; chrom; chromosome del-ins; del, deletion (whole or partial gene deletion); LOF, loss of function; ms, missense; PZ som, post-zygotic somatic mosaicism.

**Table 2:** Syndromic and Non-Syndromic Monogenic Disorders Associated with Hereditary Hypercalcaemia/PHPT

	Estimated prevalence	Gene	Chrom. Pos	Inherit.	Variant type	Genetic testing strategy
**Syndromic Forms of PHPT**						
Multiple Endocrine Neoplasia Type 1 (MEN1)	~1/30,000	*MEN1*	11q13.1	AD	ms, LOF, del^b^	Single Gene/Gene Panel (MLPA)
Multiple Endocrine Neoplasia Type 2A (MEN2A)	~1/35,000	*RET*	10q11.21	AD	ms	Single Gene/Gene Panel
Multiple Endocrine Neoplasia Type 4 (MEN4)	Rare (<1/million)	*CDKN1B^a^*	12p13.1	AD	ms, LOF	Single Gene/Gene Panel
Hyperparathyroidism Jaw Tumor (HPT-JT) Syndrome	Rare (<1/million)	*CDC73*	1q31.2	AD	ms, LOF, del^b^	Single Gene/Gene Panel (MLPA)
**Non-Syndromic Forms of PHPT including FHH**						
Familial Isolated Hyperparathyroidism (FIHP)	Uncertain	*GCM2^c^*	6p24.2	AD	ms,	Single Gene/Gene Panel
	-	*(MEN1^c^)*	11q13.1	AD	ms, LOF	Single Gene/Gene Panel (MLPA)
	-	*(CDC73^c^)*	1q31.2	AD	ms, LOF	Single Gene/Gene Panel (MLPA)
	-	*(CASR^c^)*	3q13.33-q21.1	AD	ms, LOF	Single Gene/Gene Panel
Familial Hypocalciuric Hypercalcemia Type 1 (FHH1)	~1/1-5,000	*CASR*	3q13.33-q21.1	AD	ms, LOF	Single Gene/Gene Panel
Familial Hypocalciuric Hypercalcemia Type 2 (FHH2)	Rare	*GNA11*	19p13.3	AD	ms, (if-del)	Single Gene/Gene Panel
Familial Hypocalciuric Hypercalcemia Type 3 (FHH3)	~1/13,000	*AP2S1*	19q13.32	AD	ms (Arg15)	Single Gene/Gene Panel
Neonatal Severe Hyperparathyroidism (NSHPT)	Rare	*CASR*	3q13.33-q21.1	AR/AD	ms, LOF	Single Gene/Gene Panel
**Miscellaneous Conditions Associated with Hypercalcaemia**						
Infantile Hypercalcaemia type 1	rare	*CYP24A1*	20q13.2	AR	LOF	Single gene/Gene Panel
Infantile Hypercalcaemia type 2	rare	*SLC34A1*	5q35.3	AR	LOF	Single gene/Gene Panel
Hypophosphatasia	~1/100,000	*TNSALP*	1p36.12	AR (AD)	ms, LOF	Single gene/Gene Panel
Williams-Bueren Syndrome	~1/10,000	7q11.23 microdeletion	7q11.23	de novo, AD	1.5-1.8Mb deletion	aCGH
Jansen metaphyseal chondrodysplasia (MC)	rare	*PTH1R*	3p21.31	AD	ms	Single gene/Gene Panel

a In addition to the association with MEN4, germline variants in *CDKN1B* as well as other cyclin-dependent kinase inhibitor genes (e.g., *CDKN1A, CDKN2B, CDKN2C)* have been reported in apparently sporadic parathyroid adenomas in the absence of a relevant family history such that their role as predisposition genes for hereditary PHPT remains uncertain.

b Several monogenic disorders may result from partial or whole gene deletions, which may not be detected by single-gene or gene-panel testing and may require alternate methods for detection (e.g. MLPA or aCGH), although some NGS platforms have the capability to detect these abnormalities. Loss of function (LOF) variants include nonsense mutations (i.e., resulting from a SNV introducing a premature stop codon), small insertions or deletions (indels) typically resulting in frameshift alterations, and those affected canonical splice sites resulting in aberrant transcript processing.

c FIHP represent a heterogeneous disorder whose incidence is uncertain. Approximately 15-20% of FIHP kindreds are reported to harbour *GCM2* mutations, although these variants are reported to be associated with low disease penetrance. Kindreds with FIHP have also been identified to harbour *MEN1* and *CDC73* mutations but distinguishing FIHP from the wider PHPT clinical syndromes is difficult as PHPT is frequently the first manifestation of disease, and for practical purposes such kindreds should be followed up as per the guidelines for MEN1 and HPT-JT, respectively. Although a small number of kindreds with apparent FIHP have been reported with heterozygous or homozygous inactivating *CASR* mutations, most individuals/kindreds with inactivating *CASR* mutations will have a biochemical phenotype consistent with FHH, which is not improved with parathyroid surgery.

Abbreviations: aCGH, array comparative genomic hybridization; AD, autosomal dominant; AR, autosomal recessive; chrom pos; chromosomal position; del, deletion; if-del, in-frame deletion; ms, missense; LOF, loss of function

**Table 3:** Syndromic and Non-Syndromic Monogenic Disorders Associated with Hypocalcaemia/HP

	Estimated prevalence	Gene(s) / location	Chrom. Pos	Inherit.	Variant type	Genetic testing strategy
**Non-syndromic Monogenic Hypocalcaemic disorders**						
Isolated hypoparathyroidism	Rare (<1/million)	*PTH*	11p15.3	AR/AD	ms, LOF	Single Gene/Gene Panel
Isolated Hypoparathyroidism	Unknown^a^	*GCM2*	6p24.2	AR	LOF, ms	Single Gene/Gene Panel
Isolated Hypoparathyroidism	Rare (<1/million)	*Del/Ins (SOX3)*	Xq26-27	XLR	chrom. del/ins	aCGH/other
Autosomal Dominant Hypocalcaemia Type 1 (ADH1)	~1/25,000	*CASR*	3q13.33-q21.1	AD	ms	Single Gene/Gene Panel
Autosomal Dominant Hypocalcaemia Type 1 (ADH2)	Rare	*GNA11*	19p13.3	AD	ms	Single Gene/Gene Panel
**Syndromic Hypoparathyroid disorders**						
DiGeorge Syndrome Type 1 (DGS1)	~1/4,000	*TBX1*	22q11.21	AD	del	aCGH
DiGeorge Syndrome type 2 (DGS2)	Rare (<1/million)	*NEBL*	10p13-14	AD	del	aCGH
^b^CHARGE	~1/10,000	*CHD7*	8q12.2	AD	LOF, ms	Single Gene/Gene Panel
Autoimmune Polyendocrine Syndrome type 1 (APS1)	~1/100,000	*AIRE*	21q22.3	AR	LOF, ms, del	Single Gene/Gene Panel/Del-dup
Hypoparathyroidism Deafness, Renal dysplasia (HDR)	Rare (<1/million)	*GATA3*	10p14	AD	LOF, ms	Single Gene/Gene Panel
^b^Kearns-Sayre Syndrome	~1/100,000	*Mit. gene*	NA	MIT	del	Mit. Del/dup analysis
^b^MELAS	~1/5,000	*MT-TL1 + others*	Mit. tRNA	MIT	del, LOF	Single Gene / Mit. Gene Panel
^b^Mitochondrial trifunctional protein (MTP) deficiency	~1/100,000	*HADHB*	2p23	AR	ms, del, dup	Biochemical/single gene testing^c^
Kenny-Caffey Type 1, Sanjad-Sakati syndrome	Rare^d^	*TBCE*	1q42.3	AR	LOF, ms	Single Gene/Gene Panel
Kenny-Caffey Type 2	Rare (~1/million)	*FAM111A*	11q12.1	AD	LOF, ms	Single Gene/Gene Panel
**Pseudohypoparathyroidism (PHP)**						
Pseudohypoparathyroidism Type 1a (PHP1A) / Albright’s Hereditary Osteodystrophy (AHO)	~1/100,000	*GNAS*	20q13.3	AD (IMP)^e^	LOF, ms, del,	Del-dup/single gene/MS-MLPA
Pseudopseudohypoparathyroidism (PPHP)	~1/100,000	*GNAS*	20q13.3	AD (IMP)^e^	LOF, ms, del	Del-dup/single gene/MS-MLPA
Pseudohypoparathyroidism Type 1b (PHP1B)	~1/150,000	*GNAS, STX16, NESP55*	20q13.3	AD (IMP)^e^	meth/del	Del/dup/MS-MLPA

a The incidence of isolated familial hypoparathyroidism due to *GCM2* mutations is unknown, but is reported to be the most common autosomal inherited cause of HP

b HP is a rare or occasional feature in these disorders.

c MTP deficiency is typically diagnosed on biochemical testing in the neonatal period but confirmatory genetic testing may be undertaken.

d Although Kenny-Caffey type 1 is rare, the associated Sanjad-Sakati syndrome is reported to be more common in Middle Eastern populations (prevalence ~1/10-50,000).

e The *GNAS* gene occurs within a complex imprinted locus. Maternally inherited heterozygous inactivating *GNAS* mutations are associated with PHP1A/AHO, whilst the equivalent paternally inherited variants are associated with PPHP. PHP1B is associated with methylation defects of the *GNAS* locus most commonly due to a maternally inherited deletions within the linked *STX16* gene, but also resulting from deletions involving *NESP55* or the NESP antisense (NESPAS) transcript

Loss of function (LOF) variants include nonsense mutations (i.e., resulting from a SNV introducing a premature stop codon), small insertions or deletions (indels) typically resulting in frameshift alterations, and those affected canonical splice sites resulting in aberrant transcript processing. Abbreviations: aCGH, array comparative genomic hybridization; AD, autosomal dominant; AR, autosomal recessive; chrom del-ins, chromosomal deletion-insertion; del, deletion; del-dup, deletion or duplication; IMP, imprinted locus; LOF, loss of function; MIT/mit, mitochondrial; ms, missense,; MS-MLPA, methylation-specific multiplex ligation-dependent probe amplification; meth, methylation defect; XLR, X-linked recessive.

## Data Availability

Data sharing is not applicable to this review article.
